# Kidney Transplantation Improves Survival in Lupus Nephritis With End-Stage Kidney Disease

**DOI:** 10.1016/j.ekir.2025.01.034

**Published:** 2025-02-03

**Authors:** Benoît Brilland, Jean-François Augusto, Pierre-Antoine Michel, Noémie Jourde-Chiche, Cécile Couchoud, Renal Epidemiology and Information Network (REIN) registry

**Affiliations:** 1 Service de Néphrologie-Dialyse-Transplantation, CHU Angers, Angers, France; 2 Centre de Recherche en Cancérologie et Immunologie Intégrée Nantes Angers, Inserm, CNRS, Univsité Angers, Nantes Université, SFR ICAT, Nantes, France; 3 Néphrologie et Dialyses, Département de Néphrologie, Hôpital Tenon, Assistance Publique - Hôpitaux de Paris, Paris, France; 4 Aix-Marseille Université, Centre de recherche en Cardiovasculaire et Nutrition (C2VN), Institut national de la santé des de la recherche médicale (INSERM), Institut national de recherche pour l'agriculture, l'alimentation et l'environnement (INRAE), AP-HM Centre de Néphrologie et Transplantation Rénale, Marseille, France; 5 Coordination nationale REIN, Agence de la biomédecine, Saint-Denis-La Plaine, France

**Keywords:** death, ESKD, kidney transplantation, lupus, nephritis, survival

## Abstract

**Introduction:**

Lupus nephritis (LN) is a severe complication of systemic lupus erythematosus (SLE) and is associated with high morbidity and mortality rates. Although kidney transplantation (KT) is considered the optimal treatment for end-stage kidney disease (ESKD), its survival benefit, specifically in patients with LN-induced ESKD (LN-ESKD), is not well-established. This study aimed to determine the effects of KT on the survival of a national cohort of patients with LN-ESKD.

**Methods:**

We retrospectively analyzed patients with LN-ESKD registered in the French Renal Epidemiology and Information Network (REIN) registry, who were waitlisted for KT between 2002 and 2022. KT was treated as a time-dependent variable to avoid an immortal time bias. The primary outcome was all-cause mortality, which was assessed using Kaplan-Meier analysis and adjusted Cox proportional hazards models.

**Results:**

Of the 882 patients with LN-ESKD, 636 (72%) were waitlisted for KT, and 470 (74%) received a transplant. After a median follow-up of 80 months, KT was associated with a 60% reduction in the risk of death compared with remaining on dialysis (hazard ratio [HR]: 0.40, 95% confidence interval [CI]: 0.240–0.67, *P* < 0.001), with consistent benefits across subgroups. Patient survival at 10 years was 83% for transplant recipients and 60% for nontransplant recipients (*P* < 0.001). Sensitivity analyses, after excluding recipients of living donors and patients who were inactivated from the waitlist, supported these findings. Two years after the onset of ESKD, 38% of the waitlisted patients under went transplantation. The probability of graft failure was 23% at 10 years posttransplant.

**Conclusion:**

Compared with patients who remain on dialysis, KT is associated with improved survival in patients with LN-ESKD. Early evaluation of transplant eligibility and timely referral to transplant centers are crucial for optimizing outcomes.

## Graphical abstract

**Figure:**
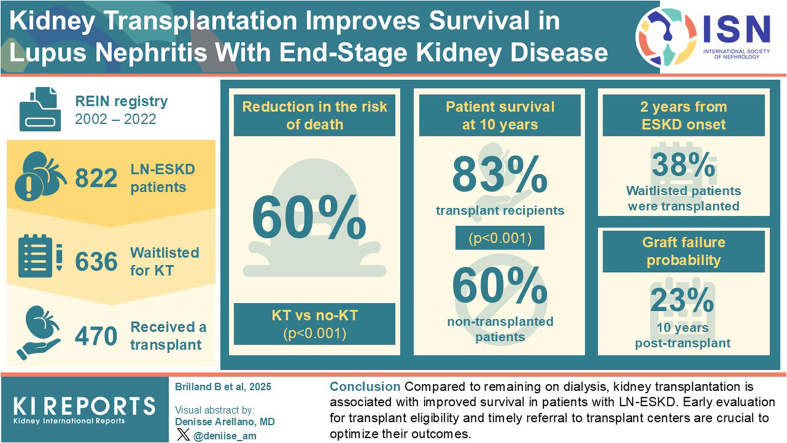

SLE has been reported to affect more than 3.4 million people worldwide and is newly diagnosed in 400,000 people each year.^1^^,^^2^ Although there is significant variation between studies, partly because of ethnic diversity, it is estimated that approximately 40% of people with SLE will develop LN over the disease course, and about 10% to 20% of people with LN will develop ESKD after 10 years.^2^^,^^3^ LN is an early or initial manifestation of SLE in approximately 70% to 80% of affected patients with LN.^4^ Beyond its impact on renal survival, LN compromises overall survival, with an estimated excess of mortality 3 to 26 times higher than that of the general population.^4^^,^^5^

KT has been found to be safe and successful in patients with LN-ESKD.^6^, ^7^, ^8^, ^9^, ^10^, ^11^ In several studies, patient and graft survival rates were comparable to those of other transplant recipients,^6^, ^7^, ^8^^,^^10^, ^11^, ^12^, ^13^ although some reports suggest lower survival outcomes in this population.^12^^,^^13^ Patients with LN-ESKD face higher risks of posttransplant complications, such as infections, cancer, thrombosis, and cardiovascular events. These risks are partly attributed to extensive pretransplant immunosuppression and, in some cases, to the presence of antiphospholipid syndrome. Furthermore, although the risk of lupus relapse decreases after transplantation, it is not entirely eliminated.^14^^,^^15^ These factors challenge the generalizability of transplantation outcomes from broader nephropathy populations to patients with LN-ESKD.

Despite these hurdles, KT remains the preferred treatment for improving overall survival in patients with LN, compared with dialysis. However, specific survival benefits of LN-ESKD remain unclear. Although small-scale studies have suggested improved survival in patients with SLE undergoing KT compared with those on dialysis^16^, ^17^, ^18^; these findings are often biased by healthier candidates being more likely to undergo transplantation. Consistent with broader studies in all-cause ESKD populations,^19^^,^^20^ the only large-scale study with a robust methodology demonstrated a 70% reduction in mortality risk for patients with LN-ESKD receiving KT compared with those on the waitlist.^21^In this study, we aimed to evaluate the impact of KT on survival in patients with LN, using data from the French National Registry of ESKD.

## Methods

### Data Collection

The REIN is a comprehensive national registry of patients who start kidney replacement therapy in France.^22^^,^^23^ Data are collected locally by each medical center and are updated prospectively annually with the help of research assistants. Data collection began in 2002 and patient follow-up continued until the earliest occurrence of KT, death, loss to follow-up, renal function recovery, or termination of the study in December 2022.

### Selection of Patients

To assess the impact of transplantation on survival, this national multicenter study included all patients who fulfilled the following criteria: (i) ESKD attributed to LN; (ii) initiated hemodialysis or peritoneal dialysis (or were preemptively transplanted) between January 1, 2002 and December 31, 2022; and (3) waitlisted for a renal transplant between January 1, 2002 and December 31, 2022. Only the first transplantation event was considered.

### Covariates and Definitions

Demographic data (age and sex); body mass index (BMI);calculated panel reactive antibody; comorbidities at baseline, including cardiovascular comorbidities (i.e., heart failure, heart rhythm disorders, peripheral arterial disease, abdominal aorta aneurysm, coronary artery disease, and stroke) and other relevant comorbidities (diabetes, respiratory insufficiency, and cancer); initial kidney replacement therapy modality (hemodialysis or peritoneal dialysis); waitlisting date (as well as removal from the waitlist and its underlying cause when applicable); transplant status; date and type (live or deceased donor) of kidney transplant; status at the end of follow-up; and, when applicable, date and cause of death were extracted from the REIN registry^23^^,^^24^ and used as covariates, exposures, or outcomes. Kidney allograft survival was assessed, and the causes of allograft loss were identified. Owing to the varying number of cardiovascular comorbidities, these were grouped together and the patients were categorized into subgroups, namely no cardiovascular comorbidity, only 1, or at least 2.

### Statistical Analysis

Continuous variables were described with mean ± SD, except for delays between events, presented as median (first–third quartiles]. Categorical variables were described as counts and percentages. Data were compared using *t* tests for continuous variables and χ^2^ test (or Fisher exact test, if necessary) for categorical variables.

The primary outcome measure was the all-cause mortality. The exposure of interest was the first KT. The date on which the patient was first waitlisted for KT was used as the start of follow-up. If waitlisting occurred before dialysis was started, the latter timepoint was considered the start of the follow-up.

We assessed transplantation as a time-dependent exposure, allocating the time spent before a renal transplant to the group that did not have transplants and the time spent after transplantation to the group that did.^19^^,^^25^ This time-dependent approach avoids immortal time bias.^26^ Kaplan Meier analysis was performed to estimate patient survival, and survival curves were compared using a log-rank test. Mortality rates per 1000 patient-years were calculated by dividing the number of deaths by the total follow-up time in years and multiplying by 1000. The 95% CI for mortality rates were estimated using Poisson’s method.

Cox proportional hazards regression analysis was conducted to identify the factors associated with mortality. Multivariate Cox regression analysis included all parameters (*P* < 0.1 in the univariate analysis). HR with 95% CI are reported. In addition, to assess the evolution of relative risk over time, we employed a cumulative time-window analysis approach. Separate Cox models were fit for cumulative 3-month increases in follow-up (e.g., baseline to month 3 and baseline to month 6, etc.) over a total period of 15 years. The follow-up period beyond each time point was censored. This method provides a dynamic estimation of the HR, thus reflecting potential variations in the effect of transplantation on the risk of death over time. Again, the 95% CI was calculated for each estimate.

Finally, to assess the differential effects across the subgroups, we calculated the *P*-values for the interaction. Subgroup analyses were performed to evaluate differences in all-cause mortality with and without transplantation according to sex, age at ESKD onset (aged < or > 40 years), year of ESKD onset (before or after 2011, the year corresponding to the median of total follow-up in the waitlisted cohort), waitlisting before or after dialysis initiation, and region of transplantation (categorized into 3 main regions).

The probabilities of access to the waitlist, access to transplantation, and graft failure were evaluated with death as a competing event using the cumulative incidence competing risk method.^27^ Cumulative incidence curves were compared using Gray’s test.

No imputation of missing data was performed. Statistical analyses were performed using R v4.3 with the *survival, survminer*, and *tidycmprsk* packages. All tests were 2-sided, and a *P*-value < 0.05 was considered statistically significant.

### Ethical Issues

The National Ethics Committee (Commission National Informatique et Liberté) approved the data collection conducted by REIN, and this study was approved by the scientific committee of REIN. The study was conducted in accordance with the principles of the Declaration of Helsinki and its amendments. Informed consent was not required to participate in the registry. Data collection was ruled out with implicit consent and with a drop-out option.

## Results

### Baseline Characteristics of All Patients With LN-ESKD

**Figure 1:**
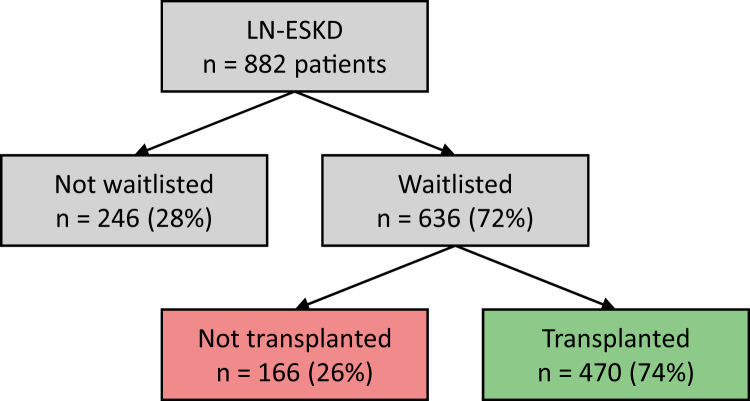
Flowchart of the study. ESKD, end-stage kidney disease; LN, lupus nephritis.

Between January 1, 2002 and December 31, 2022, 882 patients were diagnosed with LN-ESKD and registered in the French REIN Registry. Overall, 636 of 882 patients (72%) were waitlisted for KT during the study period, of whom 470 of 636 (74%) subsequently underwent KT during follow-up (Figure 1). The patients with LN-ESKD were mainly female (79%), with a mean age of 45 ± 16 years at dialysis initiation. Most patients were enrolled for hemodialysis (81%), with fewer receiving peritoneal dialysis (13%) or preemptive transplantation (6%).

In comparison to patients with LN-ESKD who were not waitlisted, waitlisted patients were younger (aged 41 ± 13 vs. 55 ± 18 years at dialysis initiation) and had fewer comorbidities (Table 1).

**Table 1:** Baseline characteristics of patients with LN-ESKD

Characteristics	ESKD *N* = 882	ESKD Not waitlisted *n* = 246	ESKD Waitlisted *n* = 636	*P*-value^a^	Waitlisted Not transplanted *n* = 166	Waitlisted Transplanted *n* = 470	*P*-value^b^
Baseline characteristics							
Female sex, *n* (%)	701 (79%)	185 (75%)	516 (81%)	0.051	136 (82%)	380 (81%)	0.8
Age							
at ESKD (yrs)	45 (16)	55 (18)	41 (13)	<0.001	46 (13)	40 (12)	< 0.001
at waitlisting (yrs)	-	-	42 (13)	-	47 (13)	40 (12)	< 0.001
at transplantation (yrs)	-	-	42 (12)	-	-	42 (12)	-
Waitlisted before dialysis initiation, *n* (%)	-	-	167 (26%)	-	35 (21%)	132 (28%)	0.078
First modality, *n* (%)				<0.001			< 0.001
Hemodialysis	712 (81%)	230 (93%)	482 (76%)		145 (87%)	337 (72%)	
Peritoneal dialysis	116 (13%)	16 (6.5%)	100 (16%)		21 (13%)	79 (17%)	
Preemptive transplantation	54 (6.1%)	0 (0%)	54 (8.5%)		0 (0%)	54 (11%)	
Region of KRT, *n* (%)				<0.001			0.5
Ile-de-France	204 (23%)	36 (15%)	168 (26%)		48 (29%)	120 (26%)	
North	287 (33%)	91 (37%)	196 (31%)		46 (28%)	150 (32%)	
South	391 (44%)	119 (48%)	272 (43%)		72 (43%)	200 (43%)	
Calculated panel reactive antibody (%)	-	-	30 (36)		38 (38)	27 (34)	0.001
Comorbidities, *n* (%)							
Not able to walk alone	54 (7.4%)	43 (20%)	11 (2.1%)	<0.001	6 (4.0%)	5 (1.4%)	0.088
Albumin level < 30 g/l	296 (42%)	115 (54%)	181 (37%)	<0.001	61 (42%)	120 (35%)	0.12
Albumin level < 36 g/l	510 (73%)	175 (82%)	335 (69%)	<0.001	109 (76%)	226 (66%)	0.033
BMI, *n* (%)				0.8			< 0.001
< 23 kg/m^2^	427 (59%)	120 (61%)	307 (58%)		73 (53%)	234 (60%)	
23–30 kg/m^2^	228 (31%)	58 (29%)	170 (32%)		40 (29%)	130 (33%)	
> 30 kg/m^2^	74 (10%)	20 (10%)	54 (10%)		26 (19%)	28 (7.1%)	
Diabetes mellitus, *n* (%)	82 (9.5%)	38 (16%)	44 (7.0%)	<0.001	17 (10%)	27 (5.9%)	0.059
Cardiovascular comorbidities, *n* (%)				<0.001			0.027
None	622 (73%)	129 (54%)	493 (80%)		120 (73%)	373 (82%)	
Only 1	145 (17%)	53 (22%)	92 (15%)		30 (18%)	62 (14%)	
At least 2	88 (10%)	55 (23%)	33 (5.3%)		14 (8.5%)	19 (4.2%)	
Cardiovascular comorbidities (detailed), *n* (%)							
Stroke/transient ischemic attack	71 (8.4%)	33 (14%)	38 (6.2%)	<0.001	17 (10%)	21 (4.7%)	0.009
Coronary artery disease	69 (8.2%)	35 (15%)	34 (5.6%)	<0.001	15 (9.3%)	19 (4.2%)	0.016
Heart failure	114 (13%)	57 (24%)	57 (9.3%)	<0.001	19 (12%)	38 (8.5%)	0.2
Rhythm disorders	56 (6.6%)	34 (15%)	22 (3.6%)	<0.001	9 (5.5%)	13 (2.9%)	0.12
Abdominal aortic aneurysm	2 (0.3%)	2 (1.1%)	0 (0%)	0.094	0 (0%)	0 (0%)	> 0.9
Peripheral artery disease	44 (5.2%)	25 (11%)	19 (3.1%)	<0.001	5 (3.1%)	14 (3.1%)	> 0.9
Respiratory insufficiency	51 (6.0%)	29 (12%)	22 (3.6%)	<0.001	10 (6.2%)	12 (2.7%)	0.039
Cancer	26 (3.3%)	18 (7.7%)	8 (1.4%)	<0.001	4 (2.5%)	4 (1.0%)	0.2
Timings and delays^c^, *n* (%)							
Time from dialysis to waitlisting (mos)	-	-	6 (0, 15)	-	10 (1, 20)	6 (0, 14)	0.008
Time from dialysis to KT (mos)	-	-	24 (11, 44)	-	-	24 (11, 44)	-
Time from waitlisting to KT (mos)	-	-	14 (4, 30)	-	-	14 (4, 30)	-
Follow-up since dialysis initiation (mos)	75 (31–131)	31 (11–74)	91 (50–147)	<0.001	46 (22–80)	110 (68–159)	< 0.001
Follow-up since waitlisting (mos)	-	-	80 (38, 133)	-	34 (10, 61)	98 (59, 146)	< 0.001
Follow-up since transplantation (mos)	-	-	77 (36, 124)	-	-	77 (36, 124)	-
Transplantation							
Donor type, *n* (%)				-			-
None	-	246 (100%)	166 (26%)		166 (100%)	-	
Deceased donor	-	-	357 (57%)		-	357 (77%)	
Living donor	-	-	107 (17%)		-	107 (23%)	
Waitlist removal	-	-	23 (3.6%)	-	23 (14%)	-	-
Outcomes, *n* (%)							
Graft failure	-	-	88 (19%)	-	-	88 (19%)	-
Death	273 (31%)	152 (62%)	121 (19%)	<0.001	51 (31%)	70 (15%)	< 0.001
During waitlisted period	-	-	-	-	46 (90%)	-	-
After waitlist removal	-	-	-	-	5 (9.8%)	-	-
During transplant period	-	-	-	-	-	47 (67%)	-
After return to dialysis	-	-	-	-	-	23 (33%)	-

BMI, body mass index; ESKD, end-stage kidney disease; KRT, kidney replacement therapy; KT, kidney transplantation; LN, lupus nephritis.

“-” means not applicable.

a Comparison between waitlisted and nonwaitlisted patients with ESKD.

b Comparison between waitlisted not transplanted and waitlisted transplanted patients.

c Timing between events and delays are shown as median [first-third quartile], in opposition to other continuous data, shown as mean (SD).

### Baseline Characteristics of Waitlisted Patients With LN-ESKD

Most waitlisted patients were female (81%), with a mean age of 42 ± 13 years when they were waitlisted for KT and a median duration of dialysis before waiting of 6 (0–15) months. Twenty percent of the patients had at least 1 cardiovascular comorbidity, 7% had diabetes, and 4% had respiratory insufficiency. Whereas 98% of the participants could walk independently, 37% had low albumin levels (< 30 g/l). In addition, 10% had a BMI > 30 kg/m^2^ and 58% were > 23 kg/m^2^.

Compared with waitlisted patients who did not receive transplants during the study period, transplanted patients were younger (aged 40 ± 12 vs. 47 ± 13 years at waitlisting) and had fewer comorbidities. Transplanted patients had lower calculated panel reactive antibody(27 ± 34 vs. 37 ± 38). Deceased donor transplantation was the predominant method (77%), and a small proportion of patients were excluded from the waitlist (*n* = 23, 14%) (Table 1). The reasons for waitlist removal are detailed in Supplementary Table S2.

### Causes of Death in Waitlisted Patients With LN

After a median follow-up of 80 (38–133)months since waitlisting, 121 patients (19%) died: 70 (15%) received a kidney transplant (including 47 [67%] during the transplant period and 23 [33%] after returning to dialysis), and 51 (31%) did not (including 46[90%] during the waitlisted period and 5 [10%] after being removed from the waitlist) (Table 1). The cause of death was unknown in 21 patients (17%) and was classified as “other” in 22 patients (18%). The proportion of deaths because of infectious causes (21% vs. 18 %), cardiovascular causes (16% vs. 20 %), and cancer (16% vs. 4 %) were not significantly different between the transplanted and nontransplanted groups (Supplementary Table S2).

### All-Cause Mortality in Waitlisted Patients With LN

**Figure 2:**
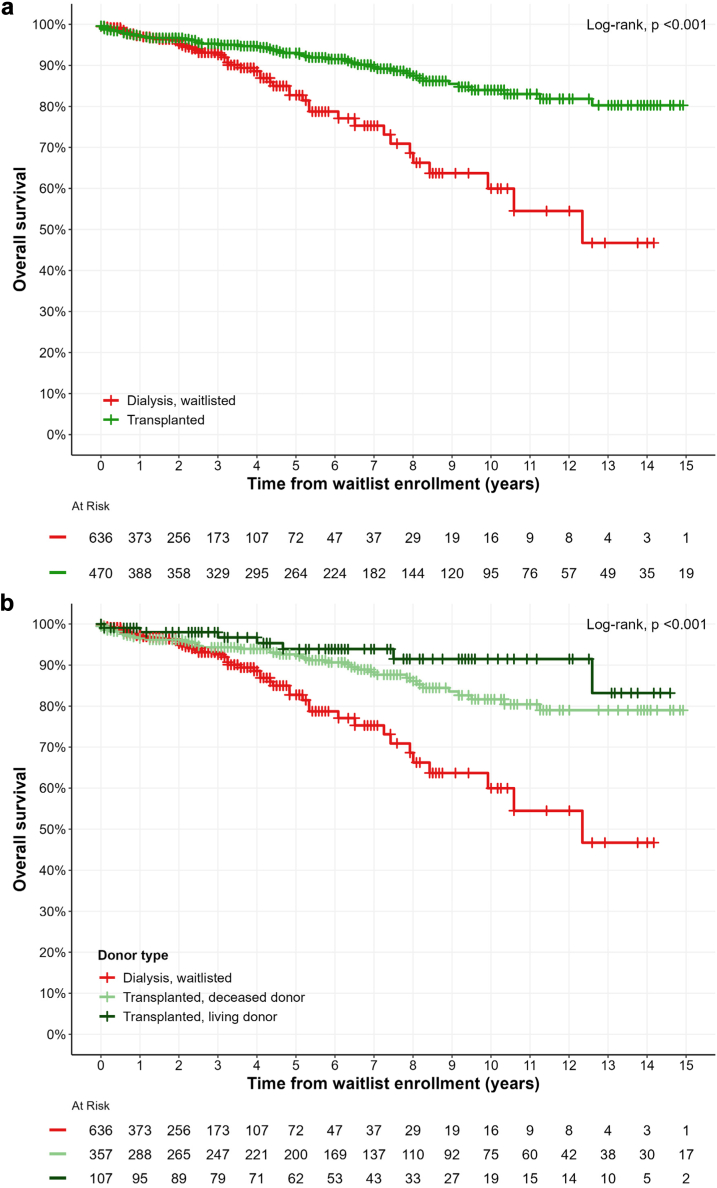
Patient survival according to transplant status among waitlisted patients with LN-ESKD. **(a) All waitlisted patients with lupus nephritis. (b) According to donor type (deceased or living donor). Benjamini-Hochberg–adjusted log-rank *P*-values: deceased donor transplantation versus no transplantation = 0.0072; living donor transplantation versus no transplantation = 0.00055;** d**eceased donor transplantation versus living donor transplantation = 0.16.** The total group size was twice that of the transplant group (once for the dialysis period and once for the transplant period).

The mortality rate among kidney transplant recipients was 16.5 (95% CI: 11.8–21.2) per 1000 patient-years, compared with 36.0 (95% CI: 26.1–46.0) per 1000 patient-years in those who did not receive transplants (*P* < 0.001, Figure 2a). This reduction in mortality was consistent across all subgroups (Table 2).

**Table 2:** All-cause mortality according to transplant status among waitlisted patients with LN-ESKD

Subgroup	*n*	Total follow-up (yrs)	Deaths	Mortality rate (/1000 person-yrs)	Mortality rate 95% CI	Unadjusted			Adjusted on age/sex			Fully adjusted^a^		
						HR	HR 95% CI	*P*-value for interaction	HR	HR 95% CI	*P*-value for interaction	HR	HR 95% CI	*P*-value for interaction
Overall	1106	4266	98	22.97	18.42–27.52									
Dialysis, waitlisted	636	1415	51	36.04	26.15–45.93	—	—		—	—		—	—	
Transplanted	470	2851	47	16.49	11.77–21.20	0.41	0.27–0.63		0.46	0.30–0.71		0.40	0.24–0.67	
Age (waitlisting)								0.92			0.99			0.54
≤ 40 yrs	518	2079	15	7.21	3.56–10.87									
Dialysis, waitlisted	284	638	7	10.98	2.85–19.11	—	—		—	—		—	—	
Transplanted	234	1441	8	5.55	1.70–9.40	0.74	0.26–2.15		0.74	0.25–2.15		0.55	0.16–1.90	
> 40 yrs	588	2187	83	37.95	29.79–46.12									
Dialysis, waitlisted	352	778	44	56.58	39.86–73.30	—	—		—	—		—	—	
Transplanted	236	1409	39	27.67	18.99–36.36	0.38	0.24–0.61		0.4	0.25–0.65		0.37	0.21–0.65	
Sex								0.25			0.39			0.33
Female	896	3437	81	23.57	18.44–28.70									
Dialysis, waitlisted	516	1180	41	34.75	24.11–45.38	—	—		—	—		—	—	
Transplanted	380	2257	40	17.72	12.23–23.22	0.46	0.29–0.73		0.5	0.31–0.80		0.45	0.26–0.78	
Male	210	829	17	20.5	10.75–30.24									
Dialysis, waitlisted	120	235	10	42.5	16.16–68.83	—	—		—	—		—	—	
Transplanted	90	594	7	11.78	3.05–20.51	0.25	0.09–0.71		0.29	0.1–0.83		0.23	0.06–0.89	
Waitlisted before dialysis initiation								0.66			0.77			0.46
No	807	3249	81	24.93	19.50–30.36									
Dialysis, waitlisted	469	1166	43	36.89	25.86–47.92	—	—		—	—		—		
Transplanted	338	2083	38	18.24	12.44–24.04	0.43	0.27–0.69		0.49	0.3–0.78		0.45		
Yes	299	1017	17	16.71	8.77–24.66									
Dialysis, waitlisted	167	250	8	32.05	9.84–54.26	—	—		—	—		—		
Transplanted	132	767	9	11.73	4.07–19.39	0.35	0.12–1.01		0.47	0.15–1.4		0.22		
Date of KRT start								0.7			0.46			0.29
Before 2011	385	2224	36	16.19	10.90–21.48									
Dialysis, waitlisted	203	526	14	26.61	12.67–40.54	—	—		—	—		—	—	
Transplanted	182	1698	22	12.96	7.54–18.38	0.44	0.21–0.91		0.44	0.21–0.92		0.33	0.13–0.82	
After 20211	721	2042	62	30.36	22.80–37.91									
Dialysis, waitlisted	433	889	37	41.62	28.21–55.03	—	—		—	—		—	—	
Transplanted	288	1153	25	21.68	13.18–30.18	0.45	0.26–0.76		0.49	0.29–0.84		0.43	0.23–0.8	
Region of KRT								0.65			0.46			0.75
Ile-de-France	288	1085	32	29.49	19.27–39.70									
Dialysis, waitlisted	168	446	16	35.9	18.31–53.49	—	—		—	—		—	—	
Transplanted	120	640	16	25.02	12.76–37.28	0.61	0.29–1.25		0.63	0.3–1.31		0.38	0.12–1.18	
North	346	1331	26	19.54	12.03–27.05									
Dialysis, waitlisted	196	396	11	27.75	11.35–44.15	—	—		—	—		—	—	
Transplanted	150	934	15	16.06	7.93–24.18	0.64	0.27–1.47		0.57	0.23–1.37		0.53	0.21–1.36	
South	472	1850	40	21.62	14.92–28.32									
Dialysis, waitlisted	272	573	24	41.88	25.12–58.63	—	—		—	—		—	—	
Transplanted	200	1277	16	12.53	6.39–18.67	0.24	0.12–0.48		0.34	0.17–0.7		0.38	0.18–0.81	

BMI, body mass index; CI, confidence interval; ESKD, end-stage kidney disease; HR, hazard ratio; KRT, kidney replacement therapy; LN, lupus nephritis.

The total group size was twice that of the transplant group (once for the dialysis period and once for the transplant period).

Regions were grouped as “North” (Bretagne, Centre-Val de Loire, Normandie, Pays de la Loire, Bourgogne-Franche-Comté, Grand Est, Hauts-de-France, La Réunion et Mayotte), “South” (Auvergne-Rhône-Alpes, Corse, Provence-Alpes-Côte d'Azur, Languedoc-Roussillon, Nouvelle-Aquitaine, Midi-Pyrénées, Guadeloupe, Martinique, Guyane) and “Ile-de-France”).

a Fully adjusted models were adjusted for age at waitlisting, sex, diabetes, BMI, and cardiovascular comorbidities (Supplementary Table S3).

Among waitlisted patients with LN, the 5-year survival rates were 93% (90%–96%) for transplant recipients and 83% (77%–89%) for nontransplant patients. At 10 years, the survival rates were 84% (79%–89%) for transplant recipients and 60% (48%–75%) for nontransplant recipients. This improved survival rate was the highest among recipients of living donor transplants compared with the nontransplanted group (*P* < 0.001, Figure 2b).

### Mortality Risk Among Waitlisted Patients With LN

The factors associated with mortality in the univariate analyses are presented in Table 3. Age at waitlisting, low BMI (< 23 kg/m^2^) and high BMI (> 30 kg/m^2^), and cardiovascular comorbidities were associated with mortality. There was a strong association between diabetes and mortality. Albumin levels > 36 g/l and KT were associated with survival.

**Table 3:** Factors associated with death among waitlisted patients with LN-ESKD

Characteristics	Univariable analysis					Multivariable analysis				*P*-value
	*n*	Deaths	HR	95% CI	*P*-value	*n*	Deaths	HR	95% CI	
Baseline characteristics										
Male sex	210	17	0.87	0.52–1.47	0.6	169	12	0.9	0.48–1.70	0.8
Age (per 1 yr increment)										
at ESKD (yrs)	1106	98	1.07	1.05–1.09	<0.001					
at waitlisting (yrs)	1106	98	1.07	1.05–1.09	<0.001	895	75	1.07	1.05–1.09	<0.001
at transplantation (yrs)	470	47	1.09	1.06–1.12	<0.001					
Waitlisted before dialysis initiation	1106	98	0.67	0.40–1.13	0.13					
First modality										
Hemodialysis	819	77	—	—						
Peritoneal dialysis	179	17	1.04	0.62–1.77	0.9					
Preemptive transplantation	108	4	0.45	0.17–1.24	0.12					
Region of KRT										
Ile-de-France	288	32	—	—						
North	346	26	0.66	0.39–1.10	0.11					
South	472	40	0.74	0.46–1.18	0.2					
cPRA (per 1% increment)	885	82	1	0.99–1.00	0.2					
Comorbidities										
Not able to walk alone^a^	16	3	2.97	0.93–9.49	0.066					
Albumin level > 30 g/l^a^	529	42	0.65	0.41–1.04	0.072					
Albumin level > 36 g/l^a^	269	14	0.44	0.24–0.78	0.005					
BMI										
23–30 kg/m^2^	300	20	—	—		297	19	—	—	
< 23 kg/m^2^	541	48	1.41	0.84–2.37	0.2	518	47	2.25	1.29–3.91	0.004
> 30 kg/m^2^	82	10	2.61	1.21–5.59	0.014	80	9	2.13	0.92–4.91	0.077
Diabetes mellitus	1086	95	1.84	0.95–3.54	0.069	895	75	1.57	0.76–3.22	0.2
Cardiovascular comorbidities										
None	866	67	—	—		710	51	—	—	
Only 1	154	17	1.36	0.80–2.32	0.3	135	16	1.45	0.81–2.58	0.2
At least 2	52	9	2.32	1.15–4.65	0.018	50	8	1.1	0.50–2.42	0.8
Cardiovascular comorbidities (detailed)^b^										
Stroke / transient ischemic attack	1059	93	1.46	0.71–3.01	0.3					
Coronary artery disease	1057	92	1.89	0.87–4.10	0.11					
Heart failure	1062	93	1.12	0.58–2.15	0.7					
Rhythm disorders	1067	93	1.65	0.67–4.06	0.3					
Abdominal aortic aneurysm	704	59	—	0.00–Inf	> 0.9					
Peripheral artery disease	1061	92	2.63	1.22–5.70	0.014					
Respiratory insufficiency	1064	94	1.91	0.77–4.71	0.2					
Cancer	968	91	—	0.00–Inf	> 0.9					
Outcomes										
Waitlist removal^c^	1106	98	1.28	0.52–3.15	0.6					
Transplantation	470	47	0.41	0.27–0.63	< 0.001	379	33	0.4	0.24–0.67	<0.001
Donor type^c^										
None	636	51	—	—						
Deceased donor	357	40	0.46	0.30–0.72	< 0.001					
Living donor	107	7	0.26	0.12–0.59	0.001					

BMI, body mass index; CI, confidence interval; cPRA, calculated Panel Reactive Antibody; ESKD, end-stage kidney disease; HR, hazard ratio; inf, infinity; KRT, kidney replacement therapy; KT, kidney transplantation; LN, lupus nephritis.

The total group size was twice that of the transplant group (once for the dialysis period and once for the transplant period).

a Albumin level and ability to walk alone were not included in the multivariate model because of an elevated number of missing values (24% and 18%, respectively).

b Individual cardiovascular comorbidities were not included in the multivariable analysis (only the grouped variable was).

c Not included in the multivariable analysis. See the Sensitivity Analysis section.

After adjustments on age at waitlisting, sex, BMI, diabetes, and cardiovascular comorbidities, KT was associated with a 60% reduction in the risk of death (HR = 0.40 [0.24–0.67], *P* < 0.001) (Tables 2 and 3). When considering donor type, after adjustments on the same variables, living donor KT was associated with a 64% reduction in the risk of death (vs. no transplantation, HR = 0.36 [0.15–0.87], *P* = 0.023) and deceased donor KT was associated with a 58% reduction (vs. no transplantation, HR = 0.42 [0.24–0.72], *P* = 0.001).

### Mortality Risk Among Subgroups of Waitlisted Patients With LN

Results were homogeneous across all analyzed subgroups, including sex, age at waitlisting (< or > 40 years), year of ESKD onset (before or after 2011), waitlisting before or after dialysis initiation, and region of transplantation. After adjustments for age at waitlisting, sex, BMI, diabetes, and cardiovascular comorbidities, KT was associated with a reduction in the risk of death, without significant interaction. The strongest effect was found for patients waitlisted before dialysis initiation (HR = 0.22 [0.05–0.9]) and male patients (HR = 0.23 [0.06–0.89]), and the weakest effect was found for younger patients (HR = 0.55 [0.16–1.9]) (Table 2 and Supplementary Figure S1).

### Instantaneous Hazard of Death

When analyzing the time-dependent instantaneous hazard after full adjustment (as described above), KT was associated with an increased risk of death in the early posttransplantation period, followed by a decreased risk in the remainder of the study (Supplementary Figure S2).

### Sensitivity Analyses

We conducted 2 sensitivity analyses to validate our primary findings. First, we excluded patients removed from the waitlist (*n* = 23) to address potential confounding factors such as patients becoming too ill for transplantation (main cause of waitlist removal, 70%; Supplementary Table S1). This did not affect the observed survival benefit (adjusted HR = 0.35 [0.21–0.61], *P* < 0.001). Second, we censored living donor transplant recipients (*n* = 107, 17%), and the survival benefit remained consistent (adjusted HR = 0.41 [0.24–0.70], *p* = 0.001).

### Kidney Allograft Survival

**Figure 3:**
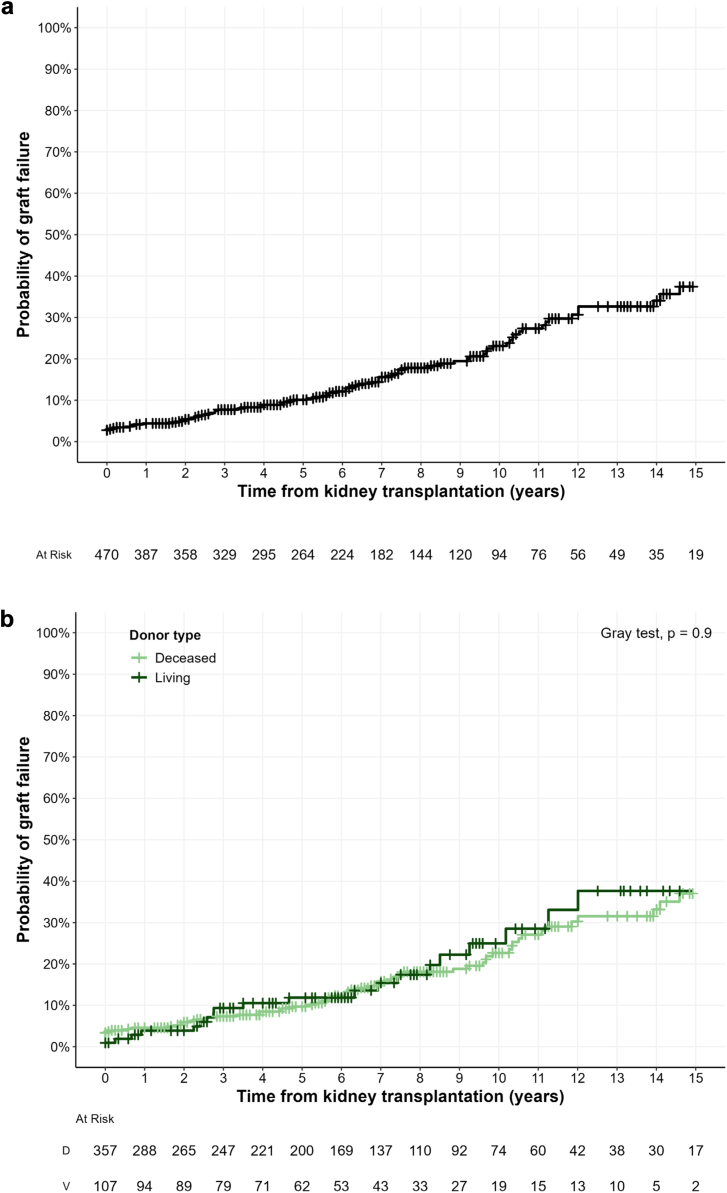
Graft survival among transplanted patients with LN-ESKD, computed with death as a competitive event. **(a) All transplanted patients with lupus nephritis. (b) According to donor type (deceased or living donor). ESKD, end-stage kidney disease**; LN, lupus nephritis.

After a median follow-up of 77 [36–124] months since transplantation, 88 patients (19%) experienced graft failure. The main cause of allograft failure was chronic rejection (*n* = 37, 42%), followed by other chronic kidney diseases (*n* = 9, 10%) and vascular complications (*n* = 9, 10%) (Supplementary Table S3). The cumulative incidence of graft failure, considering death as a competing event, was 10% (7%–13%) and 23% (18%–28%) at 5 and 10 years, respectively (Figure 3a). There was no difference in kidney allograft survival according to the donor type (deceased vs. living donors, *P* = 0.9; Figure 3b).

### Access to Waitlist and to Transplantation

Finally, we analyzed the probability of being waitlisted or transplanted, considering death as a competing event (Supplementary Figure S3). Among all patients with LN-ESKD, 47%, 63%, and 73% were waitlisted, and 14%, 28%, and 51% were transplanted at 1, 2, and 5 years after ESKD onset, respectively. Considering only waitlisted patients, 65%, 86%, and 98% were waitlisted and 19%, 38%, and 69% were transplanted 1, 2, and 5 years after the onset of ESKD, respectively. In addition, 35%, 51%, and 77% of patients underwent transplantation 1, 2, and 5 years after waitlisting, respectively.

## Discussion

This large population study, spanning 2 decades and encompassing all French patients with LN-ESKD waitlisted for KT, revealed a significant improvement in survival associated with KT. This survival benefit was consistent across the key demographics throughout the study period. However, a considerable number of waitlisted patients died before receiving a transplant, highlighting the urgent need to enhance kidney transplant accessibility for patients with LN-ESKD. Thus, these findings emphasize that early and systematic evaluation of patients with LN for transplant eligibility, ideally before ESKD onset, is critical for improving outcomes.

Our findings corroborate and extend the previous research on patients with LN-ESKD who underwent KT. The survival of patients with LN on chronic dialysis has been shown to be lower than that of other patients on dialysis,^17^^,^^18^ with a higher mortality rate from infectious and/or cardiovascular causes. Given the additional immunosuppression required, KT may be risky because of the associated risks of infection, diabetes, and hypertension. These factors limit the generalizability of the kidney transplant outcomes from other groups of patients with ESKD to patients with LN-ESKD. Our study confirms that transplantation significantly improves survival, which is consistent with data from the United States Renal Data System registry,^19^ in which deaths from infectious and cardiovascular causes have been reduced. There were no data on relapse risk reduction; however, we can assume that a modern immunosuppressive regimen, limited steroid exposure, and the inflammatory state associated with SLE also helped reduce the mortality associated with these events. Our cohort did not show a significant reduction in infectious or cardiovascular mortality. However, the increase in cancer-related mortality, which is likely not significant because of inadequate power to detect differences, may be clinically meaningful. It is important to note that waitlisted patients face higher infection-related deaths (20% in our LN-ESKD waitlisted cohort vs. 15% in all patients with ESKD regardless of its cause [data from the REIN registry]^22^), and transplanted patients face higher cancer-related deaths (16% in our LN-ESKD transplanted cohort vs. 10% in all patients with ESKD regardless of its cause [data from the REIN registry]^22^).

Taken together, these data suggest that all patients with LN should be evaluated for possible inclusion on the waiting list, as soon as estimated glomerular filtration rate goes below 20 ml/min per 1.73 m^2^, as for other kidney diseases. Early referral of patients to a kidney transplant center can improve the likelihood of preemptive transplantation, ideally with a living donor. If preemptive transplantation is not feasible, patients will accumulate time on the waitlist, potentially reducing their waiting period after the onset of ESKD. Notably, access to transplantation for all patients with LN-ESKD was very close to the all cause, patients with ESKD who were aged 18 to 59 years included in the REIN registry (2022 annual report results: 51% by 5 years).^22^

Subgroup analyses revealed that the survival benefit of transplantation was higher in male patients, who are known to experience more severe disease and higher mortality risk;^20^^,^^21^^,^^28^^,^^29^ higher in patients waitlisted before dialysis initiation, and lower in younger patients (aged < 40 years), likely because of their already low mortality on dialysis. These findings highlight the importance of tailoring strategies to improve access to transplantation, particularly in the high-risk subgroups.

This study has several strengths in terms of the data source and design. First, the REIN registry is a nationwide registry covering all patients with ESKD in France. LN was diagnosed and reported by nephrologists, thereby limiting the risk of false-positive diagnoses. Second, our methodology—time-varying exposure modeling, comorbidity adjustment, and sensitivity analyses—mitigated (but did not eliminate) biases, such as confounding or immortal time bias. In addition, we restricted our analysis to patients on the waiting list to reduce the bias associated with confounding by contraindications (people on the waiting list for KT are generally younger, healthier, and have a higher socioeconomic status^22^ than those who are not). Although residual confounding factors may still be present, we adjusted our analysis for important factors, including comorbidities, to account for variables known to influence mortality.

Our retrospective study has several limitations. The REIN registry enrolls patients when they reach ESKD, but does not include details on the history of LN. As such, we cannot address how certain factors, such as the time between LN onset, disease remission, transplantation, antibody type and titer at diagnosis or transplantation, associated antiphospholipid antibodies, exposure to immunosuppression, pretransplant events (e.g., infections, cardiovascular events, and relapses), disease activity at the time of waiting, and disease recurrence post-KT may affect outcomes. The ethnicity of patients and the use of hydroxychloroquine, both known to be associated with SLE outcomes, are unknown. In addition, treatment advances over a 2-decade study period, especially for LN management,^23^ may limit generalizability.

In conclusion, this study adds to the existing evidence supporting the significant survival benefit of KT for patients with LN-ESKD. Early referral for transplant evaluation and improved access to transplantation remain critical for improving the outcomes in this high-risk population.

## Disclosure

All the authors declared no competing interests.

## Appendix

### List of Renal Epidemiology and Information Network (REIN)

Dialysis centers participating in the registry are listed in the REIN annual report (https://www.agence-biomedecine.fr/IMG/pdf/rapport_2022_-_20241128.pdf).

## Supplementary Material

Supplementary File (PDF) **Figure S1.** Subgroup analyses regarding the risk of death among waitlisted patients with LN-ESKD. **Figure S2.** Instantaneous hazard of death over the study period. **Figure S3.** Probability of being waitlisted or transplanted. **Table S1.** Causes of waitlist removal in LN-ESKD waitlisted, not transplanted patients. **Table S2.** Causes of death among waitlisted patients with LN-ESKD. **Table S3.** Causes of allograft failure in LN-ESKD transplanted patients with LN-ESKD.
