# Supplementary material for: Kidney Transplantation Improves Survival in Lupus Nephritis With End-Stage Kidney Disease
Source: Kidney Int Rep. 2025 Feb 3;10(4):1163–74. doi: 10.1016/j.ekir.2025.01.034 (PMC12034884; doi:10.1016/j.ekir.2025.01.034)
Supplement: Supplementary File (PDF) — Figure S1. Subgroup analyses regarding the risk of death among waitlisted patients with LN-ESKD. Figure S2. Instantaneous hazard of death over the study period. Figure S3. Probability of being waitlisted or transplanted. Table S1. Causes of waitlist removal in LN-ESKD waitlisted, not transplanted patients. Table S2. Causes of death among waitlisted patients with LN-ESKD. Table S3. Causes of allograft failure in LN-ESKD transplanted patients with LN-ESKD. [file mmc1.pdf]

**Kidney transplantation improves survival in lupus nephritis with end-stage kidney disease**

Benoît Brilland[1,2], Jean-François Augusto[1,2], Pierre-Antoine Michel[3], Noémie Jourde-Chiche[4], Cécile Couchoud[5], on behalf of the REIN registry.

**Supplementary Figures and Tables legends.**

**Supplementary Figure S1. Subgroup analyses regarding the risk of death among waitlisted patients with LN-ESKD.**

Analyses are adjusted on age, sex, BMI, cardiovascular comorbidities and diabetes.

Regions were grouped as “North” (Bretagne, Centre-Val de Loire, Normandie, Pays de la Loire, Bourgogne-Franche-Comté, Grand Est, Hauts-de-France, La Réunion et Mayotte), “South” (Auvergne-Rhône-Alpes, Corse, Provence-Alpes-Côte d'Azur, Languedoc-Roussillon, Nouvelle-Aquitaine, Midi-Pyrénées, Guadeloupe, Martinique, Guyane) and “Ile-de-France”).

Abbreviations: LN, lupus nephritis; ESKD, end-stage kidney disease; KRT, kidney replacement therapy.

**Supplementary Figure S2. Instantaneous hazard of death over the study period.**

A) Raw instantaneous hazard. B) Age and sex adjusted instantaneous hazard. C) Fully adjusted (with adjustment for age at waitlisting, sex, BMI, diabetes, and cardiovascular comorbidity) instantaneous hazard.

**Supplementary Figure S3. Probability of being waitlisted or transplanted.**

Probability to access to waitlist or transplantation was computed with death as a competitive event.

Abbreviations: ESKD, end-stage kidney disease; KT, kidney transplantation; WL, waitlisting.

**Supplementary Table S1. Causes of waitlist removal in LN-ESKD waitlisted, not transplanted patients.**

**Supplementary Table S2. Causes of death in LN-ESKD waitlisted patients.**

* Comparison between the 4 categories.

** Comparison between the 12 categories.

Abbreviations: ESKD, end-stage kidney disease; LN, lupus nephritis.

**Supplementary Table S3. Causes of allograft failure in LN-ESKD transplanted patients.**

## Sup. Figure 1

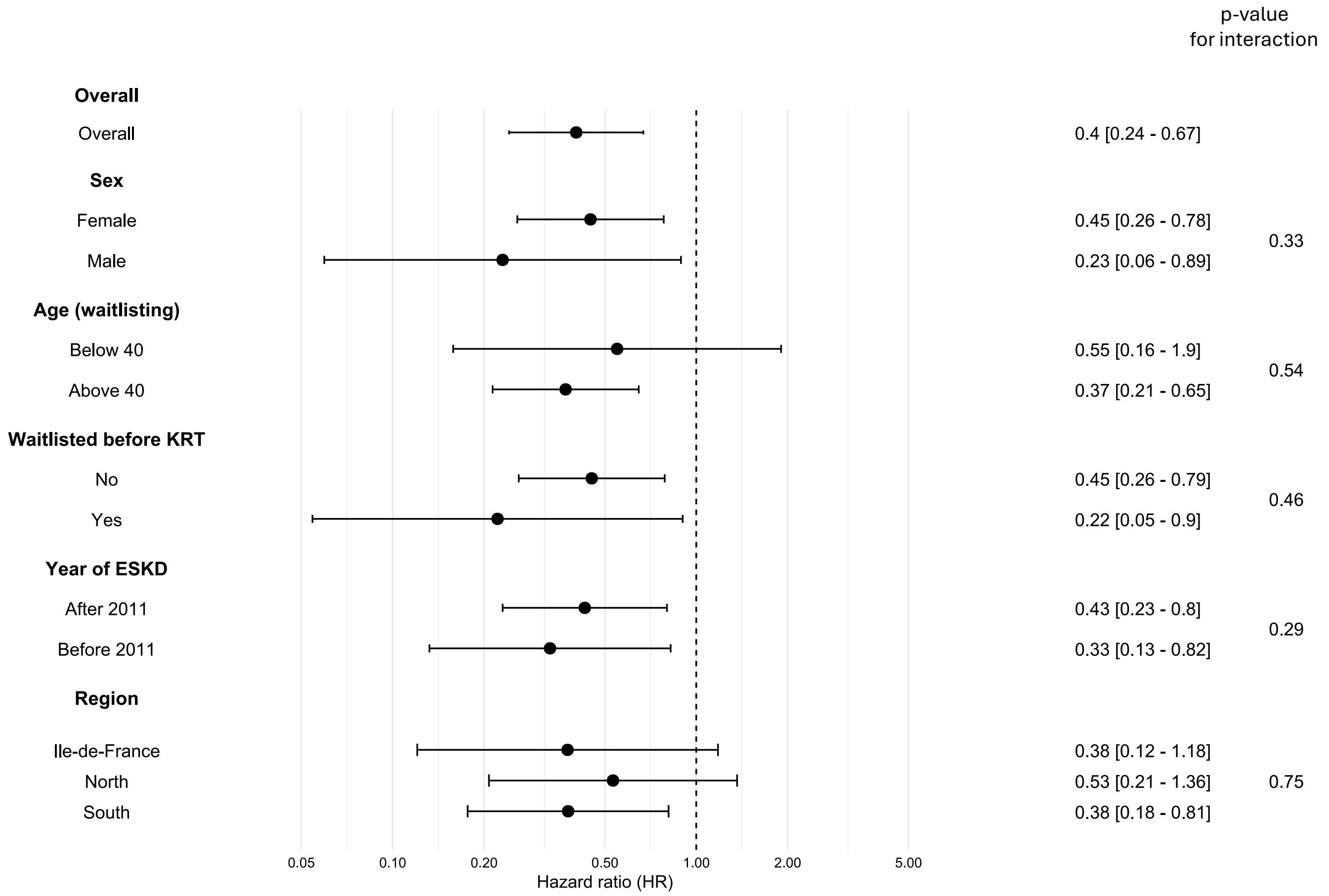

p-value
for interaction
Overall
Overall
0.4 [0.24 - 0.67]
Sex
Female
0.45 [0.26 - 0.78]
Male
0.23 [0.06 - 0.89]
0.33
Age (waitlisting)
Below 40
0.55 [0.16 - 1.9]
Above 40
0.37 [0.21 - 0.65]
0.54
Waitlisted before KRT
No
0.45 [0.26 - 0.79]
Yes
0.22 [0.05 - 0.9]
0.46
Year of ESKD
After 2011
0.43 [0.23 - 0.8]
Before 2011
0.33 [0.13 - 0.82]
0.29
Region
Ile-de-France
0.38 [0.12 - 1.18]
North
0.53 [0.21 - 1.36]
South
0.38 [0.18 - 0.81]
0.75
0.05
0.10
0.20
0.50
1.00
2.00
5.00
Hazard ratio (HR)

**Sup. Figure 2**

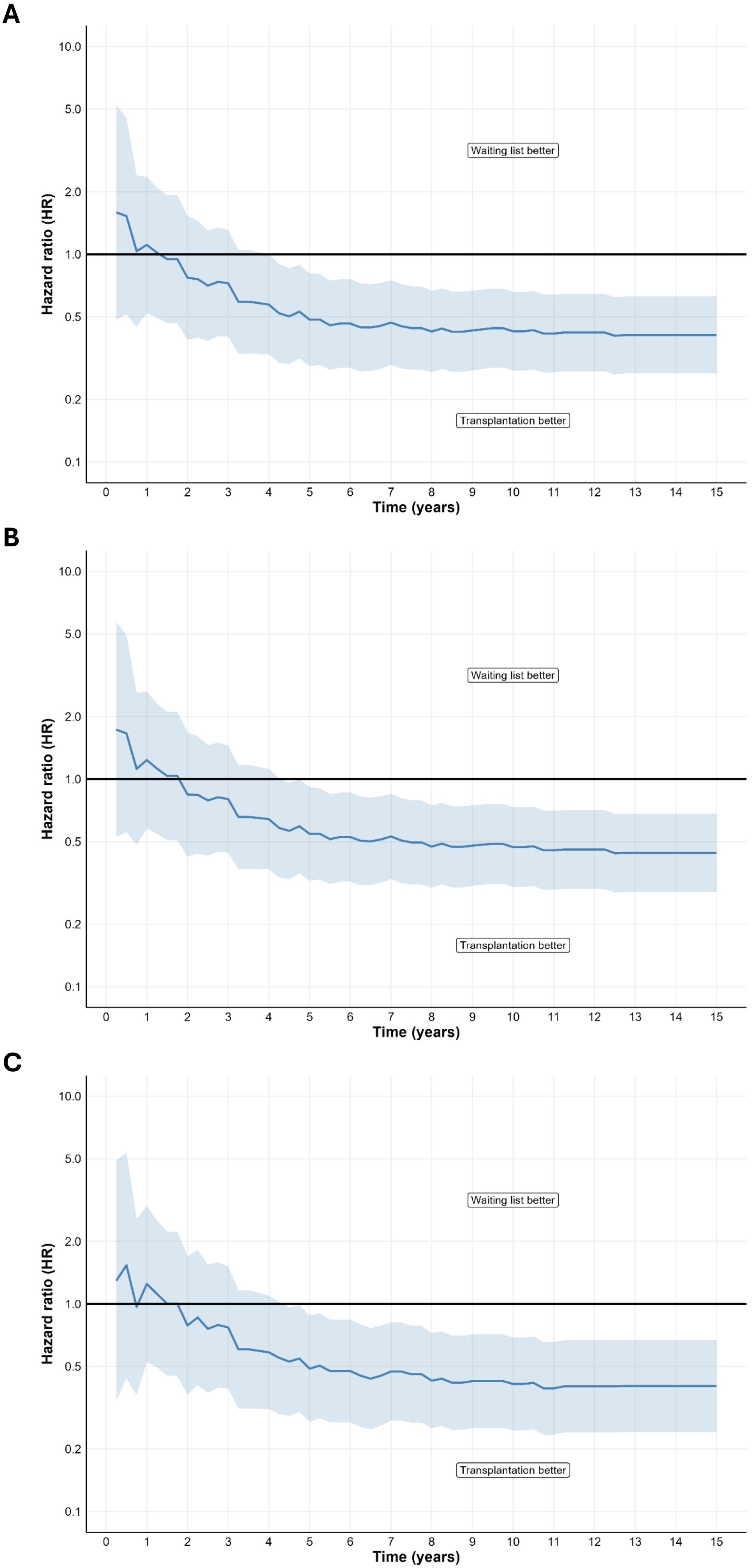

A
Hazard ratio (HR)
10.0
5.0
2.0
1.0
0.5
0.2
0.1
Waiting list better
Transplantation better
0 1 2 3 4 5 6 7 8 9 10 11 12 13 14 15
Time (years)
B
Hazard ratio (HR)
10.0
5.0
2.0
1.0
0.5
0.2
0.1
Waiting list better
Transplantation better
0 1 2 3 4 5 6 7 8 9 10 11 12 13 14 15
Time (years)
C
Hazard ratio (HR)
10.0
5.0
2.0
1.0
0.5
0.2
0.1
Waiting list better
Transplantation better
0 1 2 3 4 5 6 7 8 9 10 11 12 13 14 15
Time (years)

**Sup. Figure 3**

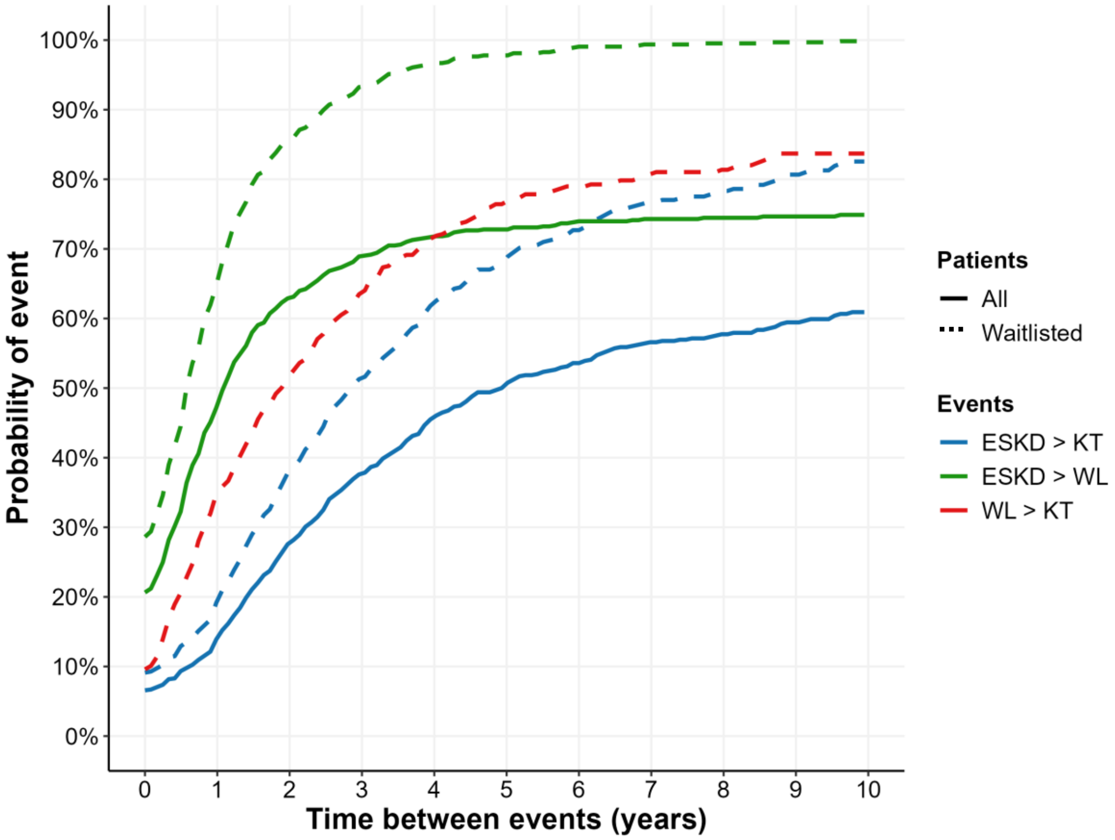
Probability of event
100%
90%
80%
70%
60%
50%
40%
30%
20%
10%
0%
0
1
2
3
4
5
6
7
8
9
10
Time between events (years)
Patients
All
Waitlisted
Events
ESKD > KT
ESKD > WL
WL > KT

**Supplementary Table S1**

| Cause of waitlist removal | Number of patients |
|---|---|
| Deterioration of clinical condition (not linked to lupus) | 14 (60.9%) |
| Patient decision | 6 (26.1%) |
| Deterioration of clinical condition (linked to lupus) | 2 (8.7%) |
| Clinical condition improvement | 1 (4.3%) |
| Total | **23 (100%)** |

**Supplementary Table S2**

| Cause of death | **Total death**, N = 121 | **Waitlisted Not transplanted**, N = 51 | **Waitlisted Transplanted**, N = 70 | **p-value** * |
|---|---|---|---|---|
| Others | 63 (52%) | 30 (59%) | 33 (47%) | 0.2 |
| Infection | 24 (20%) | 9 (18%) | 15 (21%) | |
| Cardiovascular | 21 (17%) | 10 (20%) | 11 (16%) | |
| Cancer | 13 (11%) | 2 (3.9%) | 11 (16%) | |

| **Cause of death** | **Total death**, N = 121 | **Waitlisted Not transplanted**, N = 51 | **Waitlisted Transplanted**, N = 70 | **p-value** ** |
|---|---|---|---|---|
| Other known cause | 22 (18%) | 9 (18%) | 13 (19%) | 0.2 |
| Unknown cause | 21 (17%) | 10 (20%) | 11 (16%) | |
| Rapid or unexpected death, shock without precision | 15 (12%) | 7 (14%) | 8 (11%) | |
| Cachexia | 3 (2.5%) | 2 (3.9%) | 1 (1.4%) | |
| Hyperkaliemia | 2 (1.7%) | 2 (3.9%) | 0 (0%) | |
| Infection | 24 (20%) | 9 (18%) | 15 (21%) | |
| Cerebrovascular disease | 7 (5.8%) | 4 (7.8%) | 3 (4.3%) | |
| Other cardiovascular cause | 5 (4.1%) | 1 (2.0%) | 4 (5.7%) | |
| Heart failure | 5 (4.1%) | 2 (3.9%) | 3 (4.3%) | |
| Rhythm disorder | 3 (2.5%) | 3 (5.9%) | 0 (0%) | |
| Pulmonary embolism | 1 (0.8%) | 0 (0%) | 1 (1.4%) | |
| Cancer | 13 (11%) | 2 (3.9%) | 11 (16%) | |

**Supplementary Table S3**

| Cause of allograft failure | Number of patients |
|---|---|
| Rejection | 42 (47.8%) |
| Hyperacute | 2 (2.3%) |
| Acute | 3 (3.4%) |
| Chronic | 37 (42.0%) |
| Chronic kidney disease | 9 (10.2%) |
| Vascular complications | 9 (10.2%) |
| Inobservance | 3 (3.4%) |
| Sepsis | 3 (3.4%) |
| Primarily non functioning transplant | 2 (2.3%) |
| Acute kidney injury | 2 (2.3%) |
| Lupus relapse | 1 (1.1%) |
| Heart failure | 1 (1.1%) |
| Other | 16 (18.2%) |
| Total | **88 (100%)** |
